# *IL13* gene polymorphisms among Sudanese patients with bronchial asthma: a case-control study

**DOI:** 10.22099/mbrc.2024.50865.2017

**Published:** 2025

**Authors:** Menas A. Abdalla, Hamdan Z. Hamdan

**Affiliations:** 1 Department of Pharmacology, Faculty of Pharmacy, Al-Neelain University, Khartoum, Sudan; 2 Department of Pathology, College of Medicine, Qassim University, Saudi Arabia

**Keywords:** Bronchial asthma; Gene mutation; Genetic variation; IL-13

## Abstract

Genetic polymorphisms in interleukin-13 (*IL13*) gene have been associated with asthma susceptibility in different ethnicities. We investigated the association of two polymorphisms in the *IL13* gene [rs1800925 (c.-93+487C>T), and rs20541 (p.Gln144Arg)] with asthma susceptibility among Sudanese patients. A case-control study was conducted at Al-Shaab Teaching Hospital between April and October 2022. Involving fifty asthmatic patients and fifty controls. The genotypes were determined using an allele-specific polymerase chain reaction. For rs1800925, a significant association with asthma in multivariate analysis (aOR=3.15, 95% CI: 1.13–8.76; p=0.028). The T allele was the most frequent in cases and showed a significant association with asthma (aOR=1.99, 95% CI: 1.13–3.5; p=0.016). The rs20541 did not show any association with asthma. The rs1800925 is associated with an increased risk of asthma in Sudanese patients.

## INTRODUCTION

Bronchial asthma is a common chronic respiratory disease characterized by reversible and variable degrees of chest tightness and difficulty in expiration. It is considered a complex multifactorial disease, with both genetic factors and environmental triggers contributing to its pathogenesis [1]. It has been observed that the genes susceptible to asthma are clustered on the long arm of chromosome-5, where the interleukin-13 (IL-13) is located. IL-13, secreted by type-2 helper cells (T_H_2) upon stimulation, has been linked to increased asthma susceptibility and elevated levels of immunoglobulin-E (IgE) [2]. 

Previous studies have demonstrated that the rs1800925 (c.-93+487C>T) promoter variant in the 5'–UTR is associated with asthma in Dutch and Malaysian populations [3, 4]. Another variant, rs20541 (c.431G>A) p.Gln144Arg, a missense variant that substitutes glutamine with arginine, has been associated with asthma in British and Japanese populations [5]. Little is known about the effect of these polymorphisms in association with bronchial asthma among sub-Saharan African patients, including the Sudanese population. Therefore, we investigated two *IL13* polymorphisms to determine their possible association with bronchial asthma in Sudanese patients. 

## MATERIALS AND METHODS

This case-control study was conducted in Al-Shaab Teaching Hospital in Khartoum, Sudan, between April and October 2022. The diagnosis of Asthma was based on the GINA diagnostic criteria. The cases were adult patients diagnosed with asthma and had no other comorbidities. Controls were healthy individuals without asthma. Clinical and socio-demographic characteris-tics of the participants were collected using a structured questionnaire. 

Three milliliters of blood were withdrawn from each participant and used for genomic DNA extraction. Genotyping was performed using AS-PCR. The primers and PCR conditions were adapted from a previous study with some modifications [6]. Sanger sequencing was used to validate the SNPs in IL-13. The data were analyzed using Statistical Package for Social Sciences (SPSS) version 24.0. Categorical variables were compared using chi-square test. Continuous variables were compared by student t-test. Deviation from Hardy Weinberg equilibrium (HWE) was calculated by chi-square goodness of fit test, as recommended [7, 8]. A multivariate analysis was conducted, crude and adjusted odds-ratio were calculated. The *p*-value <0.05 considered statistically significant. 

## RESULTS AND DISCUSSION

Table 1 presents the sociodemographic and clinical characteristics of the participants. The genotype frequencies of rs1800925 and rs20541 were in accordance with HWE expectations. 

**Table 1:** Sociodemographic and clinical characteristics of the participants

**Variables**	**Cases (50)**	**Controls (50)**	***p*** **-value**
**Gender **			NA
Male	29	29	
Female	21	21	
**Age, years**	38.3(14.0)	39.2(16)	0.197
**Body mass index (BMI), kg/m** ^2^	24.5(2.4)	22.4(3.7)	0.152

The genotype frequencies for rs1800925 and rs20541 in cases and controls were presented in Table 2. The multivariate analysis revealed that only genotype TT in rs1800925 is a significant risk factor for asthma, while the genotype CC was not associated with asthma. For rs20541 multivariate analysis showed that none of the genotypes were associated with asthma.

**Table 2:** Association between *IL13* polymorphisms and the risk of asthma

**Genotypes/allele**	**Cases (50)**	**Controls (50)**	**cOR (95%CI)**	**p-value**	**aOR (95%CI)**	***p*** **-value**
**rs1800925**						
CC	6(12%)	12(24%)	0.59(0.19–1.80)	0.361	0.57(0.18–1.76)	0.401
TC	26(52%)	31(62%)	Reference			
TT	18 (36%)	7(14%)	3.06(1.10–8.47)	0.030	3.15(1.13–8.76)	0.028
C Allele	0.38	0.55	Reference	-	-	-
T Allele	0.62	0.45	1.99(1.13–3.50)	0.016		
**rs20541**						
AA	15(30%)	17(34%)	0.85(0.36–2.01)	0.718	0.89(0.37–2.13)	0.797
AG	30(60%)	29(58%)	Reference	-		
GG	5 (10%)	4(8%)	1.20(0.26–4.95)	0.793	1.17(0.28–4.86)	0.822
A allele	0.60	0.63	Reference	-		
G allele	0.40	0.37	1.13(0.62–2.00)	0.662		

The major finding in this study is the significant risk association between rs1800925 and asthma development. Our finding aligns with reports from Dutch, Malaysian, and African American patients [3, 4, 9]. However, others have reported no association [10, 11]. The varying impact of this SNP across populations is not surprising, as each ethnic group appears to have a unique genetic effect. 

The rs1800925 variant is located in the 5'–UTR of the *IL13* gene. In-silico tools [12] predict that the presence of T allele allows attachment to a transcription factor named as serum response factor, unlike the wild-type C allele. Perhaps this may explain the experimental findings that show up-regulation of *IL13* gene expression in the presence of the TT genotype [13]. Notably, asthmatic patients have elevated levels of IL-13 in their blood and lung tissues [14, 15]. This high concentration of IL-13 is negatively correlated with pulmonary function test readings [16], which may explain increased antibody production, and may provide a rationale for the association with asthma. 

The second polymorphism in this study is the missense variant rs20541 (p.Gln144Arg), which showed no association with asthma. Our findings are consistent with previous reports from Egyptian and Iranian patients [17, 18]. On the contrary, other studies have reported a significant association [19, 20]. It has been reported that the presence of the A allele results in the replacement of arginine with glutamine in IL-13, leading to conformational changes and stronger interaction with its receptor [21]. This interaction increases IgE synthesis and secretion [21].

To the best of our knowledge, this is the first study to investigate *IL13* gene polymorphisms in association with asthma among Sudanese patients. Our findings revealed a significant risk association of rs1800925 with asthma development, while rs20541 was not associated with asthma in this setting. This study has some limitations that need to be considered. First, we did not quantify blood levels of IgE in the investigated groups. Second, we did not measure the expression profile of these SNPs, so no functional roles can be deduced. Therefore, further studies are warranted.
